# Effects of Taurine on Sperm Quality during Room Temperature Storage in Hu Sheep

**DOI:** 10.3390/ani11092725

**Published:** 2021-09-18

**Authors:** Liuming Zhang, Yanhu Wang, Tariq Sohail, Yan Kang, Haoyuan Niu, Xiaomei Sun, Dejun Ji, Yongjun Li

**Affiliations:** Key Laboratory for Animal Genetics & Molecular Breeding of Jiangsu Province, College of Animal Science and Technology, Yangzhou University, Yangzhou 225009, China; 18352767281@163.com (L.Z.); WYH18805274475@163.com (Y.W.); Drtariqsohail34@yahoo.com (T.S.); ky2583141771@163.com (Y.K.); nhy1101080832@163.com (H.N.); sunxiaomei@live.com (X.S.); djji@yzu.edu.cn (D.J.)

**Keywords:** taurine, Hu ram semen, room temperature, sperm quality, oxidative stress

## Abstract

**Simple Summary:**

Hu sheep sperm is highly susceptible to ROS during storage at room temperature. It is very important to use an antioxidant to ameliorate oxidative damage. Tau is an important amino acid peptide antioxidant with a wide range of biological effects. It can effectively scavenge free radicals, regulate reproductive function, improve immunity, and enhance its antioxidant capacity. However, the effects of Tau in the preservation of Hu sheep semen at room temperature are unclear. Therefore, Tau was added to Hu sheep semen preserved at room temperature to explore its effect on semen. The results showed that adding an appropriate concentration of Tau had a positive effect on Hu sheep semen preserved at room temperature; in particular, 20 mM Tau performed best.

**Abstract:**

The present study aimed to investigate whether the presence of Tau protected Hu sheep sperm from ROS stress during storage at room temperature. The semen was diluted with extender (Tris-based) at room temperature, supplemented with different concentrations of Tau (0, 10, 20, 40, 80, or 100 mM), and stored at 15 °C. Sperm quality parameters (sperm progressive motility, kinetic parameters, plasma membrane integrity rate, acrosome integrity rate, and MMP) and antioxidant parameters (ROS, MDA, SOD, CAT, and T-AOC) were evaluated during the preservation of semen. The addition of Tau, especially at a concentration of 20 mM, exerted positive effects on sperm quality parameters and antioxidant parameters compared to the sperm without Tau treatment (control group). The addition of Tau, especially at a concentration of 100 mM, exerted negative effects on sperm quality parameters and antioxidant parameters compared to the control group. Interestingly, the results indicated that the sperm acrosome integrity rate did not change during storage time. In conclusion, the addition of Tau to sperm preserved at room temperature can enhance the antioxidant ability of sperm, reduce the LPO on the 5th day, and improve the quality of semen preserved at room temperature. These results implied that Tau had potential to enhance Hu sheep sperm reproductive performance.

## 1. Introduction

The success of artificial insemination depends largely on the quality of preserved semen. Semen preservation is a technology that reduces the metabolism of sperm and prolongs the preservation time of semen after a specific treatment and preservation in the corresponding environment [1]. Storage at room temperature does not require expensive equipment, the operation is simple, and it is suitable for short-term storage of various livestock semen. The principle of room temperature preservation is to reduce the pH of the semen to a specific level through the addition of acidic substances in the extender, inhibit the metabolic activity of the sperm, and make the sperm in a reversible static state, thereby prolonging the survival time of the sperm in vitro [2]. During the preservation of semen at room temperature, with the extension of the preservation time, the sperm’s own antioxidant system cannot balance excessive reactive oxygen species (ROS). Excessive ROS will cause oxidative stress of the sperm and seriously affect the preservation effect of semen [3,4].

As the plasma membrane of sheep sperm contains a lot of polyunsaturated fatty acids (PUFAs), the ROS in semen will undergo a lipid peroxidation reaction (LPO) with the PUFAs on the plasma membrane to produce harmful substances such as Malondialdehyde (MDA) and damage the sperm DNA [5,6]. In addition, after the occurrence of LPO, the PUFAs on the plasma membrane become saturated fatty acid, which reduces or loses the fluidity of the plasma membrane [7]. The integrity of the sperm plasma membrane is the basis of sperm metabolism, capacitation, and acrosome reaction [8]. In addition, ROS will also oxidise the phosphodiester bonds and bases of DNA, destroy the stability of DNA, and cause DNA fragmentation [9]. Therefore, in order to improve the preservation of semen, eliminate excessive ROS and maintain a dynamic balance of ROS content, it is very important to add an appropriate concentration of exogenous antioxidants to the extender to inhibit or delay the production of ROS and to remove them.

Taurine (Tau) was first isolated from cattle bile, so it is also called bovine choline or taurobilin. The chemical name of Tau is β-aminoethanesulfonic acid, which is a sulfur-containing amino acid [10,11]. Tau exists in animals in a free form, has a wide range of biological effects, and can effectively scavenge free radicals, regulate reproductive function, improve immunity, and enhance its antioxidant capacity [12,13]. Tau can promote the in vitro development of embryos of cattle, pigs, rabbits, and other species. Tau is an important amino acid peptide antioxidant in the biological epididymis and reproductive tract. It not only has antioxidant effects, but also slows down cell apoptosis and regulates mitochondria [14]. Tau can also regulate the permeability of the membrane to positive ions by bidirectionally regulating the Ca^2+^ on the membrane, protect the integrity of the phospholipid layer, reduce the level of intracellular free radicals, and enhance the activity of some antioxidant enzymes [15,16]. Banday [17] found in a crossbred sheep sperm study that 40 mM Tau can improve sperm motility and reduce seminal plasma MDA after thawing. Dorado [18] found in a donkey sperm study that 60 mM Tau can improve sperm kinetic parameters preserving at 5 °C. Chhillar [19] found in a bull sperm study that 50 mM Tau can improve sperm motility and plasma membrane integrity after thawing. Aly [20] found in the study of rat sperm that Tau can reduce the adverse effects of endosulfan on sperm motility.

The effects of Tau in the preservation of Hu sheep semen at room temperature are unclear. Therefore, this study aims to test whether it can enhance sperm catalase (CAT), superoxide dismutase (SOD), total antioxidant capacity (T-AOC), functional parameters and motility parameters, reduce ROS level and MDA content, prolong the survival time of sperm, and improve the preservation quality of semen.

## 2. Materials and Methods

### 2.1. Experimental Design

Three experiments were conducted to evaluate the effects of Tau on Hu sheep sperm during room temperature. The semen source was a pool of sperm-rich ejaculates from three Hu rams, aged 2 years.

Experiment 1: effects of Tau (10, 20, 40, 80, or 100 mM) supplementation to Hu sheep semen extender on sperm motility parameters. Sperm progressive motility, straight line velocity (VSL), curvilinear velocity (VCL), average path velocity (VAP), amplitude of lateral head displacement (ALH), and average motion degree (MAD) of the control and Tau groups were evaluated at 0 d, 1 d, 3 d, 5 d, 6 d, and 7 d. Mitochondrial membrane potential (MMP) of the control and Tau groups were evaluated at 5 d.

Experiment 2: effects of Tau (10, 20, 40, 80, or 100 mM) supplementation to Hu sheep semen extender on sperm functional integrity. Plasma membrane integrity rate and acrosome integrity rate of the control and Tau groups were evaluated at 0 d, 1 d, 3 d, 5 d, and 7 d.

Experiment 3: effects of Tau (10, 20, 40, 80, or 100 mM) supplementation to Hu sheep semen extender on sperm oxidative stress parameters. ROS, SOD, T-AOC, and CAT of the control and Tau groups were evaluated at 5 d. MDA of the control and Tau groups were evaluated at 1 d, 3 d, and 5 d.

### 2.2. Preparation of Semen Extender and Semen Collection

The basic extender consisted of 15.35 g Tris, 10.00 g fructose, 8.20 g citric acid, and 250,000 IU each of penicillin and streptomycin in 500 mL distilled water; the pH was 7.47. Tau was added to the base extender at concentrations of 10, 20, 40, 80, and 100 mM, while the control was the base extender without Tau, and placed in a refrigerator at 4 °C for further use. All the above reagents were from Beijing Solarbio.

Semen samples were collected from three Hu sheep with the aid of an artificial vagina in spring. A total number of 80 ejaculates were collected from three rams every two days. The semen volume of each ram collected was from 0.8~1.5 mL every time. The semen used was milky white and had no abnormal smell. Sperm concentration reached 2.3 × 10^9^/mL. The three rams were raised in the experimental sheep farm of Yangzhou University. The rams were fed 0.2 kg concentrate/every time, twice a day, and ad libitum hay and water. Ejaculates showing >75% motility and the morphologically abnormal sperm <15% were pooled to minimize individual differences.

### 2.3. Dilution and Evaluation of Semen

The pooled fresh semen samples were split into six equal fractions in different test tubes and each test tube aliquoted 130 µL of semen. The semen was diluted 10 times at room temperature with a Tris-based extender containing different concentrations of Tau. The processed semen was stored in a 15 °C incubator and the semen was gently flipped every day.

#### 2.3.1. Sperm Motility Parameters

The preserved semen was diluted 5 times with isothermal basic extender, placed in 37 °C for 2 min, and then 1.8 µL was dropped on a special sperm counting plate and placed on a 37 °C constant temperature stage. After that, semen samples were evaluated for sperm kinetics using computer-assisted sperm analyzer (ML-608JZ II Mailang, Nanning, China).

#### 2.3.2. Plasma Membrane Integrity

The hypo-osmotic swelling test (HOST) used to evaluate the sperm plasma membrane. The hypo-osmotic solution consisted of 0.49 g Sodium citrate, 0.9 g fructose in 100 mL distilled water. A 20 µL sub-sample of preserved semen and 200 µL of hypo-osmotic solution were mixed in a test tube and incubated at 37 °C for 30 min. After shaking well, 5 µL of suspension was loaded on a slide and 200 cells with swollen and non-swollen tails were counted as sperm with membrane integrity and non-integrity, respectively, under a 400× phase-contrast microscope.

#### 2.3.3. Acrosome Integrity

Acrosome integrity was detected by Coomassie brilliant blue staining. Preparation of Coomassie Brilliant Blue Dye: We washed the container with 95% ethanol before preparation, weighed 100 mg of Coomassie Brilliant Blue G-250, and dissolved it in 50 mL of 95% absolute ethanol. At this time, it should be blue. Then, we added 100 mL of 85% phosphoric acid, which should be brown or brown-red. A 50 µL sample of preserved semen sample and 1 mL of 4% paraformaldehyde were mixed in a test tube and fixed at room temperature for 10 min. After centrifugation at 1500× *g* for 5 min, the supernatant was discarded, and 10 µL of semen taken to make a smear. After air-drying, this was stained with Coomassie Brilliant Blue dye for 30 min, then rinsed with water, and air-dried. Then, 200 cells with head stained blue and unstained blue were counted as sperm with acrosome integrity and non-integrity under a 1000× oil immersion.

#### 2.3.4. Seminal Oxidative Status Assessment

ROS level of sperm was measured using a ROS Assay Kit (Beyotime Institute of Biotechnology, Shanghai, China) according to the manufacturer’s instruction. Briefly, add 300 µL DCFH-DC working solution into the semen. Then, after staining for 30 min at 37 °C in dark conditions, the samples were washed with PBS three times. The ROS level was expressed by the fluorescence intensity. Fluorescence intensity of DCF (488 nm excitation and 525 nm emission for DCF) was detected by a multifunctional microplate reader.

The MDA content in the semen was measured using a Lipid Peroxidation MDA Assay Kit (Beyotime Institute of Biotechnology) according to the manufacturer’s instruction. Briefly, we added 0.2 mL MDA working solution into 0.1 mL sample. Then, after staining for 15 min at 100 °C, the samples were centrifuged at 1000× *g* at room temperature. Absorbance at 532 nm was detected by a multifunctional microplate reader. Finally, the results were obtained according to the standard curve.

SOD activity of sperm was measured using a Total Superoxide Dismutase Assay Kit with WST-8 (Beyotime Institute of Biotechnology) according to the manufacturer’s instruction. Prior to the required reagents, samples were centrifuged at 1000× *g* for 5 min and washed to remove the supernatant. The semen samples were lysed using the lysis buffer in the kit. The protein concentration was measured using a Detergent Compatible Bradford Protein Assay Kit (Beyotime Institute of Biotechnology) according to the manufacturer’s instruction: Add the reagent according to the procedure and detect the absorbance at 595 nm by a multifunctional microplate reader. Then, add the reagents in order according to the procedure and then stain for 30 min at 37 °C. Absorbance at 450 nm was detected by a multifunctional microplate reader. Finally, we got the result according to the formula in the instruction.

CAT activity in the semen was measured using a CAT Assay Kit (Nanjing Jiancheng Bioengineering Institute, Nanjing, China) according to the manufacturer’s instruction. The determination of CAT activity is based on the fact that the CAT decomposition reaction of H_2_O_2_ can be quickly stopped by adding ammonium molybdate. The remaining H_2_O_2_ reacts with ammonium molybdate to produce a light yellow complex, which can be measured at 405 nm. Prior to the required reagents, samples were centrifuged at 1500× *g* for 10 min and took the supernatant. Add the reagents in order according to the procedure. Finally, we got the result according to the formula in the instruction.

T-AOC in the semen was measured using a T-AOC Assay Kit (Nanjing Jiancheng Bioengineering Institute, China) according to the manufacturer’s instruction. Briefly, samples were centrifuged at 1500× *g* for 10 min and took the supernatant. Add 10 µL supernatant to the 96-well plate. Add the reagents in order according to the procedure. Absorbance at 405 nm was detected by a multifunctional microplate reader. Finally, obtain the results according to the standard curve.

#### 2.3.5. MMP

MMP of sperm was measured using a MMP Assay Kit with JC-1 (Beyotime Institute of Biotechnology) according to the manufacturer’s instruction. JC-1 is an ideal fluorescent probe widely used to detect MMP. When the MMP is higher, JC-1 accumulates in the matrix of the mitochondria. The formation of JC-1 aggregate can produce red fluorescence. When the MMP is lower, JC-1 cannot accumulate in the matrix of the mitochondria. At this time, JC-1 is a monomer and can produce green fluorescence. Prior to the required reagents, samples were centrifuged at 1500× *g* for 10 min and removed the supernatant. Add the reagents in order according to the procedure. Fluorescence intensity of JC-1 (488 nm excitation and 525 nm emission for JC-1-monomer vs. 525 nm excitation and 590 nm emission for JC-1-aggregates) was detected by a multifunctional microplate reader. Finally, just substitute the formula in the instruction. The fluorescence ratio of JC-1-aggregates (red) to JC-1-monomer (green) was the MMP.

### 2.4. Statistical Analysis

Data were analyzed using SPSS 25.0 software. The Shapiro–Wilk test was performed to detect whether the data conforms to the normal distribution. Additionally, the data show normal distribution. One-way ANOVA LSD tests were performed to assess the difference in these parameters. Significance was set at *p* ≤ 0.05 unless otherwise specified. The results are expressed as the mean ± SEM.

## 3. Results

### 3.1. Effects of Tau Supplementation on Sperm Progressive Motility

The effects of different concentrations of Tau on Hu sheep sperm progressive motility during liquid storage at 15 °C are shown in Table 1. The sperm motility of the 20, 40, and 80 mM groups was significantly higher (*p* ≤ 0.05) than that of the control group on the 3rd day. The 100 mM group significantly (*p* ≤ 0.05) reduced sperm motility. The sperm motility of the 20, 40, and 80 mM groups was significantly higher (*p* ≤ 0.05) than that of the control and 10 mM groups within 5 to 7 days. The sperm motility of the 20 mM group was significantly higher (*p* ≤ 0.05) than that of the other groups on the 7th day.

### 3.2. Effects of Tau Supplementation on Sperm Kinetic Parameters

The effects of different concentrations of Tau on Hu sheep sperm kinetic parameters during liquid storage at 15 °C are shown in Table 2. Compared with the control group, the addition of Tau did not increase (*p* > 0.05) VSL, but the 100 mM group reduced (*p* ≤ 0.05) VSL on the 3rd day. The 20 mM group had the highest VCL, VAP, and ALH, but it was not significantly (*p* > 0.05) different from the control group on the 3rd day. The MAD of the 20 mM group was significantly higher (*p* ≤ 0.05) than that of the control group on the 3rd day. The 100 mM group significantly (*p* ≤ 0.05) reduced sperm VCL, VAP, ALH, and MAD on the 3rd day. The MAD of the 20 and 40 mM groups was significantly higher (*p* ≤ 0.05) than that of the control group within 5 to 7 days. The sperm VCL, VAP, and ALH of the 20, 40, and 80 mM groups were higher (*p* > 0.05) than that of the control group on the 7th day.

### 3.3. Effects of Tau Supplementation on Sperm Plasma Membrane Integrity

The effects of different concentrations of Tau on Hu sheep sperm plasma membrane integrity during liquid storage at 15 °C are shown in Table 3. The plasma membrane integrity rate of the 80 and 100 mM groups was significantly lower (*p* ≤ 0.05) than that of the control group on the 1st day. The plasma membrane integrity rate of the 10, 20, 40, and 80 mM groups was significantly higher (*p* ≤ 0.05) than that of the control group on the 3rd day. The plasma membrane integrity rate of the 20, 40, and 80 mM group was significantly higher (*p* ≤ 0.05) than that of the control group on the 5th day. The plasma membrane integrity rate of the 20 and 40 mM groups was significantly higher (*p* ≤ 0.05) than that of the control group on the 7th day. The plasma membrane integrity rate of the 100 mM group was significantly lower (*p* ≤ 0.05) than that of the control group within 3 to 7 days.

### 3.4. Effects of Tau Supplementation on Sperm Acrosome Integrity

The effects of different concentrations of Tau on Hu sheep sperm acrosomal integrity during liquid storage at 15 °C are shown in Table 4. The acrosomal integrity rate of the 100 mM group was significantly lower (*p* ≤ 0.05) than that of the control group within 1 to 5 days. The acrosomal integrity rate of the 20, 40, and 80 mM group was significantly higher (*p* ≤ 0.05) than that of the control group on the 3rd day. The acrosomal integrity rate of the 20 and 40 mM groups was significantly higher (*p* ≤ 0.05) than that of the control group within 5 to 7 days.

### 3.5. Effects of Tau Supplementation on Sperm ROS Content

**Figure 1:**
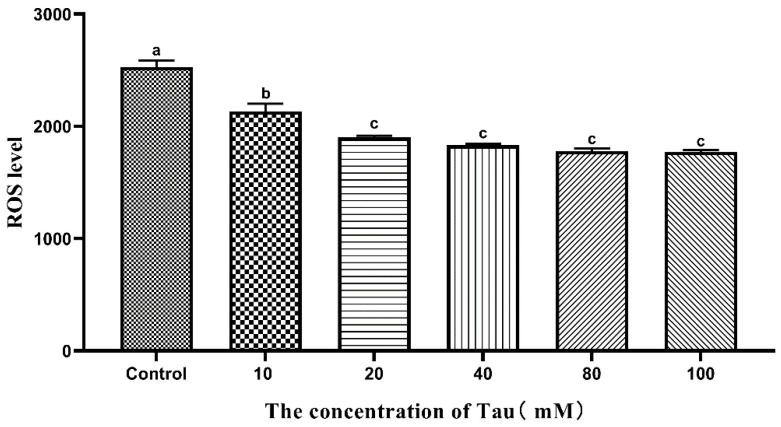
Effects of different concentrations of Tau stored at room temperature on the production of ROS in the sperm on the fifth day (n = 10). Note: Letter difference means significant difference (*p* ≤ 0.05), but the same letter means no significant difference (*p* > 0.05).

The effects of different concentrations of Tau on Hu sheep sperm ROS content during liquid storage at 15 °C are shown in Figure 1. The sperm ROS level of the 10 mM group was significantly lower (*p* ≤ 0.05) than that of the control group on the 5th day. The ROS level of the 20, 40, 80, and 100 mM groups was significantly lower (*p* ≤ 0.05) than those of the control and 10 mM groups on the 5th day.

### 3.6. Effects of Tau Supplementation on Semen MDA Content

**Figure 2:**
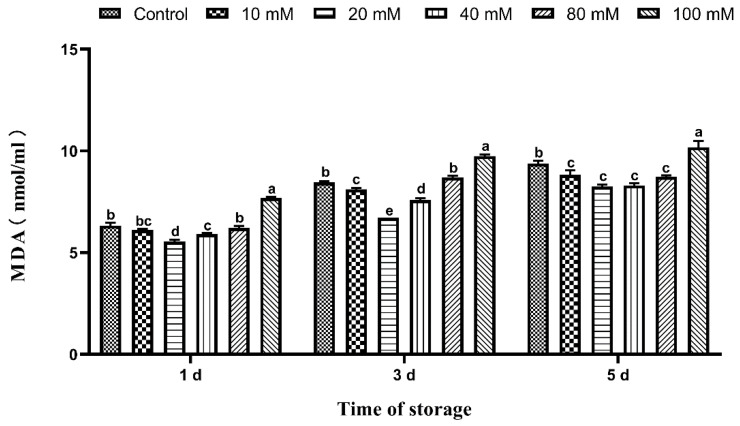
Effects of different concentrations of Tau stored at room temperature on the production of MDA in the semen (n = 10). Note: Letter difference means significant difference (*p* ≤ 0.05), but the same letter means no significant difference (*p* > 0.05).

The effects of different concentrations of Tau on Hu sheep semen MDA content during liquid storage at 15 °C are shown in Figure 2. The MDA content of semen in the 20 and 40 mM groups was significantly lower (*p* ≤ 0.05) than that of the control group on the 1st day. The MDA content of the 100 mM group was significantly higher (*p* ≤ 0.05) than that of the control group on the 1st day. The MDA content of the 10, 20, and 40 mM group was significantly lower (*p* ≤ 0.05) than that of the control group on the 3rd day. The MDA content of the 100 mM group was significantly higher (*p* ≤ 0.05) than that of the control group on the 3rd day. The MDA content of the 10, 20, 40, and 80 mM group was significantly lower (*p* ≤ 0.05) than that of the control group on the 5th day. The MDA content of the 100 mM group was significantly higher (*p* ≤ 0.05) than that of the control group on the 5th day.

### 3.7. Effects of Tau Supplementation on Sperm SOD Activity

**Figure 3:**
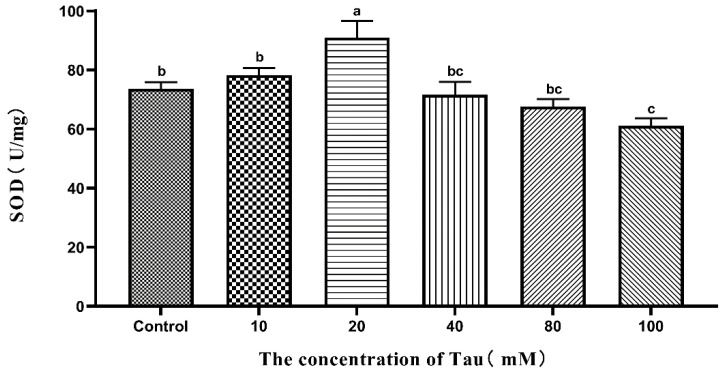
Effects of different concentrations of Tau stored at room temperature on SOD activity of the sperm on the fifth day (n = 10). Note: Letter difference means significant difference (*p* ≤ 0.05), but the same letter means no significant difference (*p* > 0.05).

The effects of different concentrations of Tau on Hu sheep sperm SOD activity during liquid storage at 15 °C are shown in Figure 3. The SOD activity of the 20 mM group was significantly higher (*p* ≤ 0.05) than that of the other groups on the 5th day. The SOD activity of the 100 mM group was significantly lower (*p* ≤ 0.05) than that of the control group and the 10 mM group on the 5th day.

### 3.8. Effects of Tau Supplementation on Semen CAT Activity

**Figure 4:**
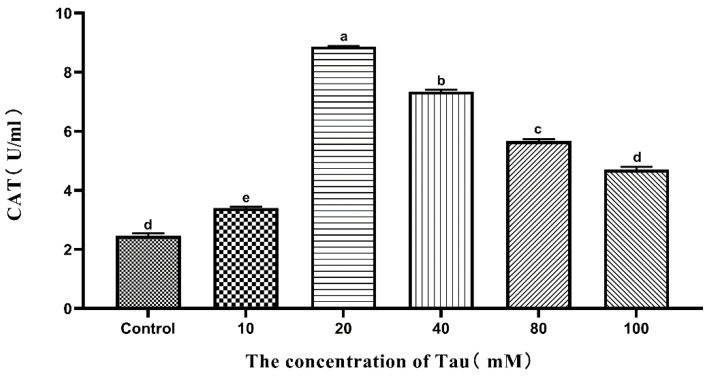
Effects of different concentrations of Tau stored at room temperature on CAT activity in the semen on the fifth day (n = 10). Note: Letter difference means significant difference (*p* ≤ 0.05), but the same letter means no significant difference (*p* > 0.05).

The effects of different concentrations of Tau on Hu sheep semen CAT activity during liquid storage at 15 °C are shown in Figure 4. The semen CAT activity of the 10, 20, 40, and 80 mM group was significantly higher (*p* ≤ 0.05) than that of the control group on the 5th day, and the 20 mM group had the highest CAT activity.

### 3.9. Effects of Tau Supplementation on Semen T-AOC

**Figure 5:**
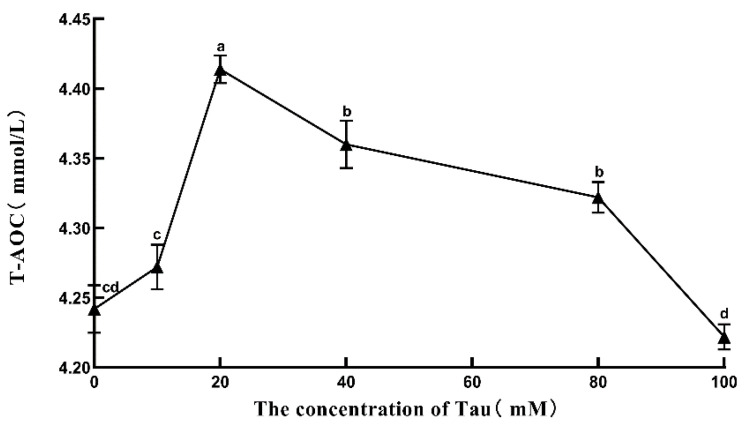
Effects of different concentrations of Tau stored at room temperature on T-AOC in the semen on the fifth day (n = 10). Note: Letter difference means significant difference (*p* ≤ 0.05), but the same letter means no significant difference (*p* > 0.05).

The effects of different concentrations of Tau on Hu sheep semen T-AOC activity during liquid storage at 15 °C are shown in Figure 5. With the increase in Tau concentration, T-AOC in the semen of Hu sheep stored at room temperature showed a trend of first rising and then falling. The semen T-AOC of the 20 mM group was the highest, and was significantly higher (*p* ≤ 0.05) than that of the control group on the 5th day. The T-AOC of the 100 mM group was lower (*p* > 0.05) than that of the control group on the 5th day.

### 3.10. Effects of Tau Supplementation on Sperm MMP

**Figure 6:**
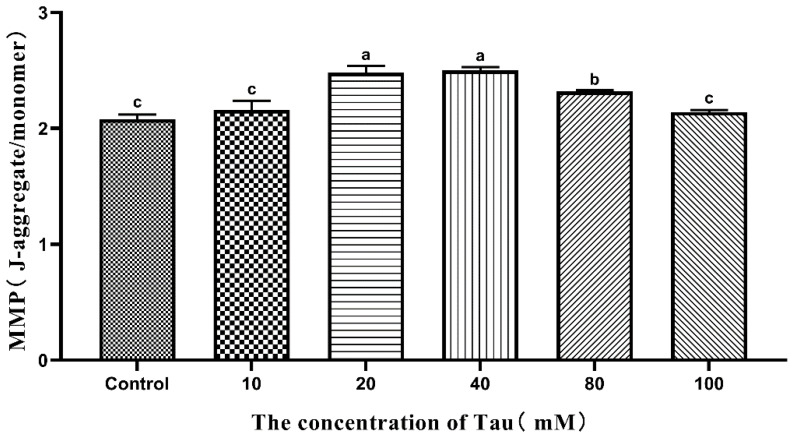
Effects of different concentrations of Tau stored at room temperature on MMP of the sperm on the fifth day (n = 10). Note: Letter difference means significant difference (*p* ≤ 0.05), but the same letter means no significant difference (*p* > 0.05).

The effects of different concentrations of Tau on Hu sheep sperm MMP during liquid storage at 15 °C are shown in Figure 6. The MMP of sperm in the 20, 40, and 80 mM group was significantly higher (*p* ≤ 0.05) than that of the control group and the 10 mM group on the 5th day. The MMP of the 40 mM group was the highest, but it was not significantly (*p* > 0.05) different from the 20 mM group.

## 4. Discussion

Semen preservation at room temperature has the advantages of simple operation, less damage to sperm, and lower investment cost. However, the semen is easily damaged by free radicals such as ROS during storage at room temperature, which shortens the survival time of sperm. Therefore, it is very important to add effective antioxidants to the semen extender. The effects of Tau on Hu sheep semen stored at room temperature are unclear. Therefore, this study aims to detect the motility parameters such as sperm progressive motility and VSL, functional integrity such as plasma membrane integrity rate and acrosome integrity rate, and oxidative stress parameters such as ROS, MDA, SOD, CAT, T-AOC, and MMP. The effects of different concentrations of Tau on the preservation of Hu sheep semen at room temperature were analyzed, and the most suitable concentration of Tau was obtained by screening.

ROS includes superoxide anion (·O^2−^), hydroxyl radical (·OH) and hydrogen peroxide (H_2_O_2_). There are four main sources of ROS in semen. First, when there is inflammation in the male animal’s reproductive system, there will be activated white blood cells in the ejaculated semen, which will then produce ROS; second, dysplastic sperm will also produce ROS; third, the mitochondrial electron transport chain complex I and III; in the process of transferring electrons, it leaks and reacts with O^2^ in the semen to produce H_2_O_2_; and fourth, when there are microorganisms in the collected semen, a certain amount of ROS will also be produced [21,22]. Sperm has its own antioxidant system, such as CAT, SOD, and glutathione peroxidase (GPX) in sperm and semen. When the ROS content is low, the system can maintain its balance, but when the ROS exceeds the physiological level, its own antioxidant system cannot balance the excessive ROS, which damages the sperm structure and reduces the quality of semen. Low concentration of ROS has certain benefits to sperm. It plays a certain role in the occurrence of sperm acrosome reaction, super-activation movement, capacitation, and sperm-egg combination [23]. However, too much ROS will cause oxidative stress of sperm, which will damage to the sperm structure such as plasma membrane and DNA [24,25]. These effects will seriously affect the preservation of semen. Therefore, adding exogenous antioxidants to semen can effectively alleviate this problem.

In this study, adding an appropriate concentration of Tau can alleviate the oxidative damage during the storage of Hu sheep semen at room temperature and improve the quality of semen preservation. This result is consistent with the results of Fote [26] in the frozen semen of cattle, and the results of Mustafa [27] in the frozen semen of goats. Bucak [28] found in an Akkaraman sheep sperm study that 25 mM Tau can significantly improve sperm CAT, but there is no significant difference in other parameters such as MDA and GSH after thawing. Ljaz [29] found in a stallion sperm study that Tau can improve sperm motility parameters preserved at 5 °C. Lone [30] found in cryopreserved crossbred ram semen study that 40 mM Tau significantly reduces the MDA in seminal plasma compared to the idebenone and resveratrol treatments. Rostami [31] found in a cryopreserved Iranian Afshari ram semen study that 25 mM Tau did not improve semen quality any better than 2 mM vitamin E. Rather [32] found in a ram semen study that 25 mM Tau can improve sperm motility parameters and plasma membrane integrity compared to vitamin C. Tau has the functions of providing nutrients for the central nervous system, protecting the liver, promoting embryonic development, and improving vision. Studies have proved that Tau is a cytoprotective agent with broad study prospects, which can protect the integrity of cell membranes, confirming that Tau can protect biological systems and protect them from oxygen free radical damage [10]. It may be based on these functions of Tau to enhance the antioxidant capacity of sperm, inhibit LPO, and then protect the structure of sperm.

In this study, within 1 to 7 days of semen storage, the concentration of 100 mM reduced sperm progressive motility, the other concentrations increased sperm progressive motility and the concentration of 20 mM reached the highest. The change of sperm progressive motility showed a trend of rising first and then falling. On the one hand, it may be as the high concentration of Tau changes the osmotic pressure of the extender, affects the permeability of the sperm membrane, destroys the sperm structure, and reduces sperm progressive motility [33]. On the other hand, it may be as the high concentration of Tau may be toxic to and damage sperm, and caused the excessive activation of antioxidant enzymes and mitochondria, which affected the physiological state of the sperm [34]. This trend is also consistent with the change trend of the plasma membrane integrity rate in this study. High concentration of Tau changed the permeability of the sperm plasma membrane and reduced the sperm plasma membrane integrity rate. The change in the acrosome integrity rate in this study did not significantly decrease over time, possibly as Tau has little effect on the acrosome structure. At a concentration of 20 mM, the T-AOC, SOD, and CAT are the highest, and the change process of MDA content is the opposite. This may be as when 20 mM Tau is added, the resistant ability to LPO is the strongest, and meanwhile the activity of antioxidant enzymes is the highest [35]. On the other hand, the structure of the sperm plasma membrane may change with the increase in Tau concentration, which has a certain impact on the LPO reaction, as the sperm plasma membrane is rich in PUFAs [36]. The MMP is the driving force for mitochondria to produce ATP, which provides energy for the movement of sperm and ensures the movement performance of sperm [37]. When the sperm is in a normal state, the MMP is higher, but when the sperm cells undergo apoptosis, the MMP is significantly reduced [38]. This study found that at a concentration of 20 mM Tau, the MMP is in a higher state, which is conducive to the maintenance of sperm MMP. This is consistent with the results of Partyka [39] in the frozen storage of chicken semen. The addition of 1 mM Tau at an appropriate concentration can increase the MMP of chicken sperm after thawing.

## 5. Conclusions

In conclusion, the results of this experiment show that when 20 mM Tau is added to the extender, sperm progressive motility, plasma membrane integrity, T-AOC, antioxidant enzymes, and MMP are significantly higher than those of the control group throughout the preservation period. Comprehensive indicators of all aspects show that the concentration of 20 mM Tau is the best for the preservation of Hu sheep semen at room temperature, which can effectively alleviate the oxidative stress damage caused by ROS, prolong the survival time of sperm, and improve the quality of semen preservation.

## Figures and Tables

**Table 1:** Effects of different concentrations of Tau on Hu ram sperm progressive motility stored at room temperature (n = 10).

Time of Storage (d)	Progressive Motility (%)					
	Control	10 mM	20 mM	40 mM	80 mM	100 mM
0	86.09 ± 1.24	85.93 ± 1.24	86.26 ± 0.75	85.94 ± 1.18	86.17 ± 1.28	86.13 ± 1.12
1	80.92 ± 1.39	80.04 ± 1.23	81.70 ± 1.16	81.28 ± 1.86	81.31 ± 0.65	76.72 ± 2.16
3	67.79 ± 0.79 ^d^	69.31 ± 0.90 ^cd^	74.41 ± 0.92 ^a^	72.00 ± 0.68 ^ab^	70.48 ± 0.39 ^bc^	63.35 ± 1.06 ^e^
5	52.93 ± 0.71 ^de^	54.64 ± 0.23 ^d^	65.49 ± 0.67 ^a^	60.75 ± 0.41 ^b^	58.11 ± 0.67 ^c^	51.64 ± 0.57 ^e^
7	24.09 ± 0.43 ^cd^	25.59 ± 0.25 ^c^	34.79 ± 0.74 ^a^	30.80 ± 0.35 ^b^	29.68 ± 0.48 ^b^	23.67 ± 0.61 ^d^

Note: Letter difference means significant difference (*p* ≤ 0.05), but the same letter means no significant difference (*p* > 0.05).

**Table 2:** Effects of different concentrations of Tau on Hu ram sperm kinetic parameters stored at room temperature (n = 10).

Index	Time of Storage (d)	Control	10 mM	20 mM	40 mM	80 mM	100 mM
VSL (μm/s)	0 d	38.47 ± 1.48	38.24 ± 0.52	35.92 ± 0.65	36.06 ± 0.74	36.59 ± 0.10	36.85 ± 0.33
	1 d	35.14 ± 0.83	33.22 ± 0.87	33.88 ± 0.19	34.63 ± 1.02	33.72 ± 0.74	34.62 ± 0.33
	3 d	32.40 ± 0.56 ^a^	30.86 ± 0.56 ^ab^	32.25 ± 0.70 ^a^	31.65 ± 0.81 ^ab^	31.91 ± 0.69 ^ab^	29.25 ± 1.49 ^b^
	5 d	28.92 ± 1.63	30.45 ± 0.89	30.75 ± 0.49	30.85 ± 0.51	27.48 ± 0.63	28.51 ± 2.53
	7 d	23.11 ± 0.73	20.60 ± 0.52	22.14 ± 0.31	22.20 ± 1.19	21.78 ± 0.41	21.92 ± 1.05
VCL (μm/s)	0 d	68.04 ± 4.13	65.85 ± 1.64	64.09 ± 2.58	65.07 ± 1.00	63.53 ± 2.77	65.85 ± 0.92
	1 d	63.51 ± 0.89	60.16 ± 1.88	60.99 ± 1.15	62.02 ± 0.83	61.29 ± 1.45	62.17 ± 1.39
	3 d	63.16 ± 1.01 ^a^	60.89 ± 0.64 ^ab^	64.41 ± 0.63 ^a^	63.23 ± 1.11 ^a^	63.41 ± 1.41 ^a^	56.94 ± 2.75 ^b^
	5 d	57.15 ± 2.65	61.94 ± 2.10	62.83 ± 0.68	62.77 ± 0.24	52.75 ± 1.99	55.61 ± 7.30
	7 d	39.09 ± 1.35 ^ab^	35.62 ± 1.22 ^b^	39.89 ± 2.07 ^ab^	40.14 ± 1.47 ^ab^	40.42 ± 0.84 ^ab^	41.89 ± 2.00 ^a^
VAP (μm/s)	0 d	48.11 ± 2.92	46.56 ± 1.16	45.32 ± 1.83	46.01 ± 0.71	44.92 ± 1.95	46.56 ± 0.65
	1 d	44.91 ± 0.63	42.54 ± 1.33	43.13 ± 0.82	43.85 ± 0.58	43.33 ± 1.02	43.96 ± 0.98
	3 d	44.66 ± 0.71 ^a^	43.06 ± 0.46 ^ab^	45.55 ± 0.44 ^a^	44.71 ± 0.78 ^a^	44.84 ± 1.00 ^a^	40.26 ± 1.94 ^b^
	5 d	40.42 ± 1.87	43.80 ± 1.48	44.43 ± 0.49	44.39 ± 0.17	37.30 ± 1.41	39.32 ± 5.16
	7 d	27.64 ± 0.96 ^ab^	25.18 ± 0.87 ^b^	28.21 ± 1.47 ^ab^	28.38 ± 1.04 ^ab^	28.58 ± 0.59 ^ab^	29.62 ± 1.42 ^a^
ALH (μm)	0 d	19.93 ± 1.21	19.29 ± 0.48	18.77 ± 0.76	19.06 ± 0.29	18.61 ± 0.81	19.29 ± 0.27
	1 d	18.60 ± 0.26	17.62 ± 0.55	17.86 ± 0.34	18.16 ± 0.24	17.95 ± 0.42	18.21 ± 0.41
	3 d	18.50 ± 0.29 ^a^	17.83 ± 0.19 ^ab^	18.87 ± 0.18 ^a^	18.52 ± 0.32 ^a^	18.57 ± 0.41 ^a^	16.68 ± 0.81 ^b^
	5 d	16.74 ± 0.77	18.14 ± 0.61	18.40 ± 0.20	18.39 ± 0.07	15.45 ± 0.58	16.29 ± 2.14
	7 d	11.45 ± 0.40 ^ab^	10.43 ± 0.36 ^b^	11.68 ± 0.61 ^ab^	11.76 ± 0.43 ^ab^	11.84 ± 0.25 ^ab^	12.27 ± 0.59 ^a^
MAD (°/s)	0 d	113.99 ± 2.79	104.76 ± 9.97	100.01 ± 6.39	110.36 ± 3.32	97.39 ± 15.22	113.28 ± 10.41
	1 d	64.92 ± 0.62	67.73 ± 4.37	69.02 ± 2.80	75.19 ± 10.97	78.67 ± 1.08	71.13 ± 2.95
	3 d	57.78 ± 2.89 ^b^	66.97 ± 2.17 ^ab^	71.98 ± 5.74 ^a^	67.02 ± 2.59 ^ab^	68.03 ± 3.21 ^ab^	58.33 ± 1.44 ^b^
	5 d	49.78 ± 2.28 ^cd^	53.41 ± 1.08 ^bc^	63.79 ± 4.16 ^a^	58.37 ± 2.35 ^ab^	55.78 ± 1.19 ^bc^	42.19 ± 2.41 ^d^
	7 d	22.69 ± 2.36 ^c^	25.86 ± 1.54 ^bc^	27.62 ± 0.93 ^b^	32.83 ± 0.98 ^a^	32.46 ± 1.10 ^a^	24.43 ± 1.22 ^bc^

Note: Letter difference means significant difference (*p* ≤ 0.05), but the same letter means no significant difference (*p* > 0.05).

**Table 3:** Effects of different concentrations of Tau on the integrity rate of plasma membrane of the Hu ram sperm stored at room temperature (n = 10).

Time of Storage (d)	Plasma Membrane Integrity (%)					
	Control	10 mM	20 mM	40 mM	80 mM	100 mM
0	73.38 ± 1.04	75.14 ± 0.58	74.39 ± 0.35	72.53 ± 0.61	73.41 ± 0.28	72.71 ± 0.20
1	65.58 ± 0.89 ^a^	65.14 ± 0.39 ^a^	65.37 ± 0.18 ^a^	63.07 ± 0.91 ^ab^	62.20 ± 0.72 ^b^	50.61 ± 1.18 ^c^
3	56.38 ± 0.82 ^c^	61.66 ± 1.68 ^ab^	64.91 ± 0.57 ^a^	61.86 ± 0.73 ^ab^	60.37 ± 0.53 ^b^	50.18 ± 1.50 ^d^
5	47.29 ± 1.90 ^d^	49.79 ± 0.43 ^cd^	57.08 ± 0.48 ^a^	54.03 ± 0.45 ^b^	50.42 ± 0.12 ^c^	42.64 ± 0.86 ^e^
7	42.59 ± 0.93 ^c^	44.21 ± 0.73 ^c^	53.88 ± 0.08 ^a^	47.76 ± 0.71 ^b^	43.15 ± 0.35 ^c^	36.88 ± 0.83 ^d^

Note: Letter difference means significant difference (*p* ≤ 0.05), but the same letter means no significant difference (*p* > 0.05).

**Table 4:** Effects of different concentrations of Tau on acrosome integrity rate of the Hu ram sperm stored at room temperature (n = 10).

Time of Storage (d)	Acrosomal Integrity (%)					
	Control	10 mM	20 mM	40 mM	80 mM	100 mM
0	95.17 ± 1.35	95.72 ± 1.01	93.49 ± 0.26	93.91 ± 0.25	93.60 ± 0.83	94.20 ± 0.62
1	92.60 ± 0.32 ^a^	91.05 ± 0.33 ^bc^	93.10 ± 0.23 ^a^	90.57 ± 0.17 ^c^	92.09 ± 0.69 ^ab^	87.39 ± 0.65 ^d^
3	85.76 ± 0.85 ^d^	87.27 ± 0.33 ^c^	91.74 ± 0.43 ^a^	90.12 ± 0.46 ^b^	87.95 ± 0.08 ^c^	83.14 ± 0.42 ^e^
5	83.93 ± 0.08 ^d^	85.25 ± 0.31 ^bc^	88.93 ± 0.36 ^a^	85.89 ± 0.23 ^b^	84.23 ± 0.22 ^cd^	81.97 ± 0.59 ^e^
7	79.66 ± 0.93 ^cd^	81.62 ± 0.80 ^bc^	85.88 ± 0.32 ^a^	83.28 ± 0.42 ^b^	81.06 ± 1.09 ^bcd^	78.92 ± 0.27 ^d^

Note: Letter difference means significant difference (*p* ≤ 0.05), but the same letter means no significant difference (*p* > 0.05).

## Data Availability

All data sets collected and analyzed during the current study are available from the corresponding author on reasonable request.
